# Attitudes toward COVID-19 vaccination during the state of emergency in Osaka, Japan

**DOI:** 10.1371/journal.pone.0279481

**Published:** 2022-12-30

**Authors:** Satomi Odani, Shihoko Koyama, Yuichi Katsumi, Isao Miyashiro

**Affiliations:** 1 Cancer Control Center, Osaka International Cancer Institute, Osaka, Japan; 2 Health Promotion Division, Department of Public Health and Medical Affairs, Osaka Prefectural Government, Osaka, Japan; Faculty of Medicine, National University of Mexico (UNAM), MEXICO

## Abstract

**Background:**

COVID-19 vaccination for general population started on April 12, 2021, in Osaka, Japan. We investigated public attitudes toward vaccination and associated factors of vaccine hesitancy during the third state of emergency.

**Methods:**

An internet-based, self-reported, cross-sectional survey was conducted in June 2021, using the smartphone health app for residents of Osaka aged ≥18 years. Respondents were asked about their attitudes toward COVID-19 vaccine. Responses “Don’t want to receive vaccines” or “Don’t know” were defined as vaccine hesitancy (vs. “Received [1^st^ dose]”, “Received [2^nd^ dose]”, or “Want to receive vaccines”). Multivariable Poisson regression analysis was conducted to examine the associations between hesitancy and population characteristics.

**Results:**

23,214 individuals (8,482 men & 14,732 women) were included in the analysis. Proportions that answered “Received (1^st^ dose)”, “Received (2^nd^ dose)”, “Want to receive vaccines”, “Don’t want to receive vaccines”, “Don’t know”, and “Don’t want to answer” were 14.6%, 3.8%, 70.6%, 4.3%, 6.1%, and 0.5% among men; and 11.3%, 6.0%, 64.9%, 6.2%, 11.0%, and 0.6% among women. Factors associated with vaccine hesitancy included being a woman (aPR = 1.33; 95%CI = 1.23–1.44), age 18–39 (aPR = 7.00; 95%CI = 6.01–8.17) and 40–64 years (aPR = 4.25; 95%CI = 3.71–4.88 vs. 65+ years), living alone (aPR = 1.19; 95%CI = 1.08–1.30 vs. living with 3+ members), non-full-time employment and unemployment (aPRs ranged 1.12 to 1.49 vs. full-time employment), cardiovascular diseases/hypertension (aPR = 0.72; 95%CI = 0.65–0.81), and pregnancy (women of reproductive age only) (aPR = 1.35; 95%CI = 1.03–1.76).

**Conclusions:**

Most respondents expressed favorable attitudes toward COVID-19 vaccination while hesitancy was disproportionately high in certain populations. Efforts are needed to ensure accessible vaccine information resources and healthcare services.

## Introduction

Coronavirus disease 2019 (COVID-19) has evolved into a global public health threat. Besides non-pharmaceutical measures (e.g. social distancing, movement restrictions, promoting personal hygiene), vaccines are expected to play a significant role in ending the COVID-19 pandemic by establishing herd immunity [1, 2].

Osaka, the third most populated prefecture (8.8 million) [3] in Japan, experienced one of the worst surges of COVID-19 infections in the nation. During the fourth wave of the pandemic (March-June 2021), daily infections and deaths marked 1,260 and 55, respectively, which were the highest record then of the prefecture [4, 5]. On April 25, 2021, the Japanese government declared to place Osaka under the third state of emergency through June 20, 2021. The COVID-19 vaccination for general population started on April 12, 2021, in several prefectures in Japan including Osaka and is currently ongoing throughout the nation as of November 2021 [6].

As getting vaccinated is a personal choice, the decision-making process is affected by a variety of intrapersonal, interpersonal, and social contexts. People’s health beliefs including perceived barriers and perceived benefits are considered as key determinants of COVID-19 vaccine hesitancy, and these are directly associated with modifiable or nonmodifiable factors such as gender, education, age, geographical locations, occupation, marital status, and race/ethnicity [7]. Vaccine hesitancy, or “*delay in acceptance or refusal of vaccination despite availability of vaccination services*” [8] is a major challenge to the establishment of herd immunity. A cross-sectional study conducted during Feb 2021 (when vaccines were available only for medical workers) reported that the vaccine hesitancy rate was 11.3% among Japanese adults, citing fears of side effects as the top reason [9]. As the national vaccination program now covers the general population, monitoring public attitudes toward vaccination is key to planning, implementing, and evaluating the rollout strategies and interventions to ensure equal access to vaccines.

In light of the above, objectives of the present study are to 1) assess public attitudes toward COVID-19 vaccine and 2) examine prevalence and associated factors of vaccine hesitancy using large-scale data.

## Methods

### Data and study population

An internet-based, self-reported, cross-sectional survey was conducted during June 1–20, 2021, using the smartphone health app “*Asmile*”. The app was launched in 2019 as part of the Osaka Health and Fitness Support Project and is available for all residents of Osaka aged 18 years or older [10]. Upon downloading the app, users provide a copy of documents to verify their identity. The users are also asked to provide a web-based informed consent that their personal information will be anonymized and used for the purpose of informing and improving the public health policies administered by the Osaka prefectural government or municipalities. With the app, the users are encouraged to keep a record of their daily physical activities and respond to surveys to earn points to apply for lotteries. Detailed information about *Asmile* are reported elsewhere [11].

The study population of the present study was individuals aged 18 years or older residing in Osaka prefecture. The survey questionnaire was developed by the Osaka prefectural government in collaboration with the Osaka International Cancer Institute. Web-based questionnaires were distributed within the smartphone app *Asmile* to all users to investigate the changes in health behaviors and attitudes during the third state of emergency in Osaka. Of approximately 246,000 users as of June 2021, 23,460 individuals responded to the survey. After excluding 246 individuals whose basic demographic information (e.g. sex, age, place of residence) unverified, a total of 23,214 respondents (8,482 men and 14,732 women; age range 18–92 years) were included in the analyses. The study was approved by the Institutional Review Board of the Osaka International Cancer Institute (approval number: 20102). The data were deidentified before use.

### Attitudes toward COVID-19 vaccination

Respondents were asked “Free-of-charge COVID-19 vaccine rollout has started. Have you already received the COVID-19 vaccine? If not, do you want to receive it?” Response categories included “Received (1^st^ dose)”, “Received (2^nd^ dose)”, “Want to receive vaccines”, “Don’t want to receive vaccines”, “Don’t know”, and “Don’t want to answer”. Vaccine hesitancy was defined as either of the responses “Don’t want to receive vaccines” or “Don’t know”. Those who answered “Don’t know” likely intended to wait for some time to see how the vaccine rollout went or simply had not considered taking the vaccine at the time of survey. Thus, they were assumed to delay decision-making and intake of the vaccine, meeting the aforementioned definition of vaccine hesitancy [8].

### Respondents’ characteristics

The assessed population characteristics included sex (man/woman), age (18-39/40-64/65+ years old), number of household members (1/2/3/4+ including self), employment status (full-time/part-time/contractor/self-employed/unemployed/other), presence of morbidities or any health conditions that are listed as potential risk factors for severe illness from COVID-19 infection (diabetes, cardiovascular disease/hypertension, chronic kidney disease, chronic liver disease, respiratory disease, cancer, immunodeficiency, neuropathy or neuromuscular disease, severe motor and intellectual disabilities, sleep apnea syndrome, obesity, and pregnancy) [12].

### Statistical analysis

Descriptive analyses were conducted to assess public attitudes toward COVID-19 vaccination and the prevalence of vaccine hesitancy. Multivariable Poisson regression analysis was conducted to examine the associations between vaccine hesitancy and population characteristics by excluding individuals who answered “Don’t want to answer” to the vaccine attitude question (N = 128). All abovementioned variables except pregnancy were included in the main model. A separate model was fitted for women of reproductive age (18–49 years) only to control for pregnancy and all other covariates. Variance inflation factors were computed for all independent variables in the models to examine correlations, and all values were confirmed to be below 5.0. All analyses were performed using R version 4.1.0.

## Results

Of the 23,214 respondents, the proportions of man and woman were 36.5% (N = 8,482) vs. 63.5% (N = 14,732), respectively, and individuals aged 18–39 years accounted for 10.3% (N = 2,397). The respondents were biased toward women and the middle-aged compared to the sampling source (i.e. all Asmile users) (39.4% men, 60.6% women, and 19.8% aged 18–39 years) [13] or the entire adult population of Osaka (47.4% men, 52.6% women) [3].

**Fig 1:**
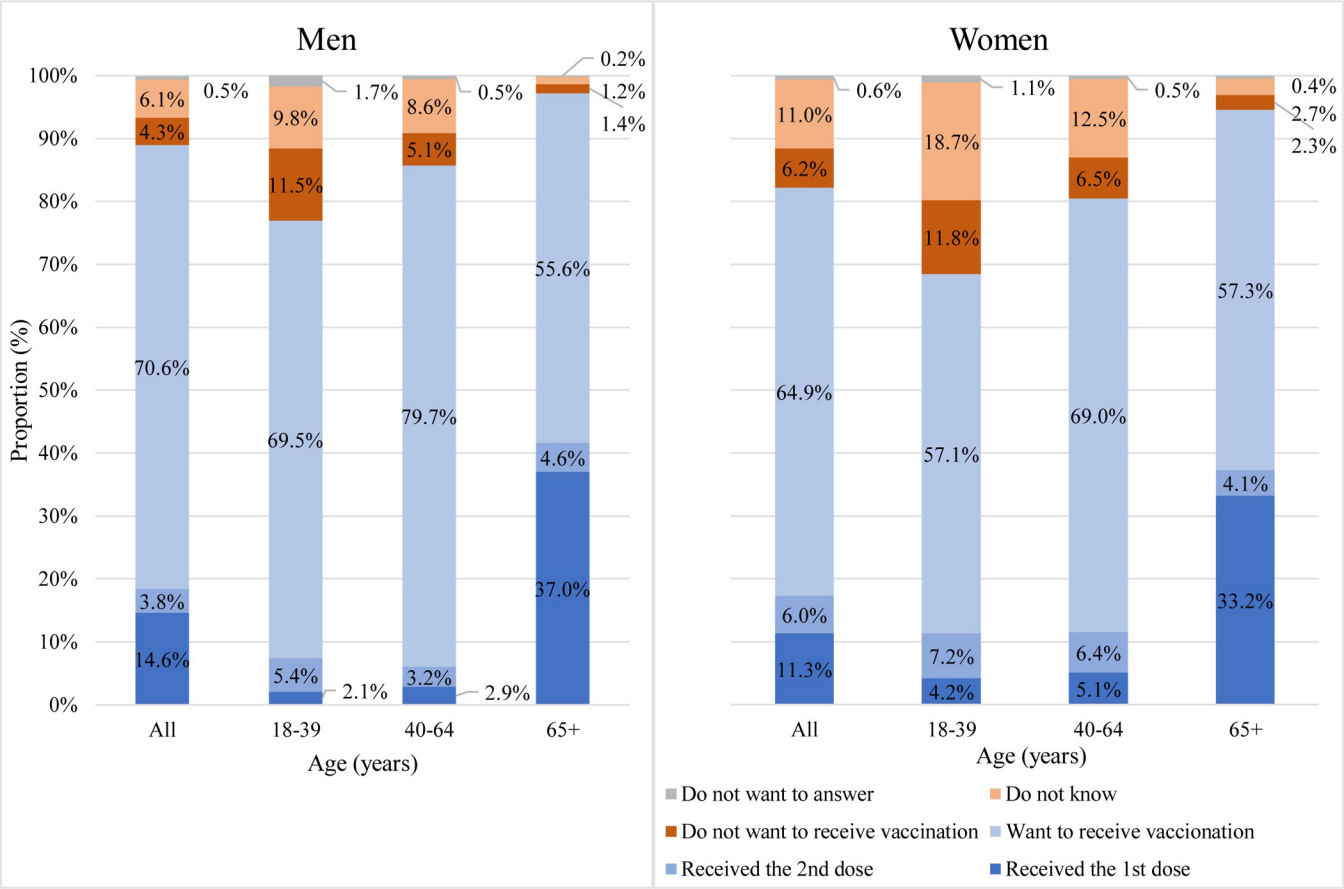
Public attitudes toward COVID-19 vaccination, Osaka, Japan, June 2021. **Note:** Data collection was conducted during June 1–20, 2021 with residents of Osaka prefecture aged ≥18 years (n = 23,214; 8,482 men & 14,732 women) through the smartphone health app “*Asmile*”. Respondents were asked “Free-of-charge COVID-19 vaccine rollout has started. Have you already received the vaccine? If not, do you want to receive it?”.

To the question asking about the attitudes toward COVID-19 vaccination, the proportions that answered “Received (1^st^ dose)”, “Received (2^nd^ dose)”, “Want to receive vaccines”, “Don’t want to receive vaccines”, “Don’t know”, and “Don’t want to answer” were 14.6%, 3.8%, 70.6%, 4.3%, 6.1%, and 0.5% among men overall; and 11.3%, 6.0%, 64.9%, 6.2%, 11.0%, and 0.6% among women overall with substantial variations across age groups (Fig 1).

14.7% of overall respondents reported vaccine hesitancy with higher prevalence among women than men (17.2% vs. 10.4%) (Table 1). By age, the prevalence ranged from 3.9% among those aged 65+ years to 28.1% among younger adults aged 18–39 years. By employment status, the highest prevalence of vaccine hesitancy was observed among contractors (23.5%) followed by the self-employed (18.4%): the lowest prevalence was seen among unemployed individuals (10.2%). According to the reported health conditions, prevalence of vaccine hesitancy was lowest among those who had diabetes (6.3%) and highest among pregnant women (33.6%).

**Table 1:** Prevalence and associated factors of COVID-19 vaccine hesitancy, Osaka, Japan, June 2021.

		Population distribution	Vaccine hesitancy	
Characteristic	Category	N (%)	Prevalence (%)	aPR (95% CI)
Overall		23214 (100.0)	14.7	-
Sex	Man	8482 (36.5)	10.4	Ref.
	Woman	14732 (63.5)	17.2	**1.33 (1.23–1.44)**
Age	18–39	2397 (10.3)	28.1	**6.99 (5.99–8.15)**
	40–64	14572 (62.8)	17.2	**4.27 (3.73–4.90)**
	65+	6245 (26.9)	3.9	Ref.
Number of household members	1	3821 (16.5)	17	**1.20 (1.09–1.31)**
(including self)	2	8853 (38.2)	12.1	1.00 (0.92–1.09)
	3	5582 (24.1)	15.7	1.03 (0.94–1.12)
	4+	4958 (21.3)	16.6	Ref.
Employment status	Full-time	7278 (31.2)	15.9	Ref.
	Part-time	5263 (22.7)	15.8	**1.12 (1.03–1.22)**
	Contractor	637 (2.7)	23.5	**1.53 (1.32–1.77)**
	Self-employed	1843 (8.0)	18.4	**1.48 (1.33–1.66)**
	Unemployed	6809 (29.4)	10.2	**1.20 (1.09–1.32)**
	Other	1384 (6.0)	17.3	**1.49 (1.32–1.69)**
Presence of health conditions (vs. "No")	Diabetes	1298 (5.6)	9.3	0.94 (0.79–1.12)
	Cardiovascular diseases/Hypertension	4165 (18.0)	7.9	**0.73 (0.65–0.81)**
	Chronic kidney disease	299 (1.3)	11.0	0.96 (0.70–1.32)
	Chronic liver disease	302 (1.3)	11.0	0.99 (0.72–1.35)
	Respiratory disease (excluding COVID-19)	597 (2.6)	15.2	1.11 (0.92–1.33)
	Cancer	295 (1.3)	10.8	0.90 (0.65–1.25)
	Immunodeficiency	386 (1.7)	16.6	1.10 (0.88–1.37)
	Neuropathy/Neuromuscular disease	382 (1.6)	16.5	1.17 (0.93–1.47)
	Severe motor and intellectual disabilities	36 (0.2)	22.2	1.26 (0.70–2.26)
	Sleep apnea syndrome	611 (2.6)	13.3	1.18 (0.97–1.44)
	Obesity (BMI≥30)	1152 (5.0)	16.4	1.12 (0.98–1.28)
Pregnancy (vs. "No")	Yes	119 (0.5)	33.6	**1.35 (1.03–1.76)**

**Abbreviations:** aPR = adjusted prevalence ratio; CI = confidence interval.

**Note:** Prevalence ratios in **bold types** were statistically significantly lower/higher than 1.00. Data collection was conducted during June 1–20, 2021 with residents of Osaka prefecture aged ≥18 years through the smartphone health app “*Asmile*”. Respondents were asked “Free-of-charge COVID-19 vaccine rollout has started. Have you already received the vaccine? If not, do you want to receive it?” Vaccine hesitancy was defined as either of the responses “Don’t want to receive vaccine” or “Don’t know”. Multivariable Poisson regression analysis was conducted to examine the associations between vaccine hesitancy and population characteristics. To examine the association between vaccine hesitancy and pregnancy, a separate model was fitted for women of reproductive age (18–49 years).

Multivariable analysis revealed that women were more likely to report vaccine hesitancy than men (adjusted prevalence ratio [aPR] = 1.33; 95% confidence interval [CI] = 1.23–1.44). Individuals aged 18–39 years and 40–64 years had 6.99 times (95%CI = 5.99–8.15) and 4.27 times (95%CI = 3.73–4.90) higher likelihoods of reporting hesitancy, respectively, than older individuals aged 65+ years. Compared to respondents living in a household of 4+ members, those living alone were more likely to report vaccine hesitancy (aPR = 1.20; 95%CI = 1.09–1.31). With full-time workers as the referent, all other types of employees or the unemployed had increased likelihood of vaccine hesitancy. The highest aPR was observed among contractors (aPR = 1.53; 95%CI = 1.32–1.77) followed by unclassified (“other”) individuals (aPR = 1.49; 95%CI = 1.32–1.69) and the self-employed (aPR = 1.48; 95%CI = 1.33–1.66). Respondents who reported having cardiovascular diseases or hypertension had lower likelihood of vaccine hesitancy (aPR = 0.73; 95%CI = 0.65–0.81) compared to those who did not have the disease. Among women, pregnant individuals had 1.35 (95%CI = 1.03–1.76) times higher likelihood of vaccine hesitancy.

## Discussions

During June 1–20, 2021, in Osaka, over 80% of respondents in both sexes reported having received at least one does of COVID-19 vaccine or intending to receive one. Vaccine hesitancy was reported by 14.7% of overall respondents with higher likelihood among women, and younger, non-full-time working or unemployed, or pregnant individuals. The present study provides a profile of public attitudes toward COVID-19 vaccination to inform the ongoing rollout strategies.

Aligned with findings from previous assessments [9, 14–17], we found that attitudes toward COVID-19 vaccination varied across sex and age groups. This disparity may partially be described by the beliefs among younger individuals that they will not get infected or become seriously ill from COVID-19 [9]. The prevalence of vaccine hesitancy found in the present study is comparable to those of other countries (reported to be 13–29%) [17] and that from the previous study of Japanese adults (11.3%) [9]. While a majority of respondents across Osaka had already received or intended to receive COVID-19 vaccines, efforts are still required to build confidence, convenience, and complacency in a COVID-19 vaccine to achieve the optimal inoculation level for all population groups. Herd immunity is achievable when a majority of the population has gained immunity. Although there are ongoing debates over the required doses and threshold, several studies have suggested that at least 60–70% of the population should be appropriately vaccinated [18–20]. The move to begin administering booster shots (third dose) of COVID-19 vaccine from December 1^st^, 2021 has been approved by the Japanese health ministry [21]. As the number of individuals who actually receive a COVID-19 vaccine may be lower than those who claim they intend to do so, continued surveillance is warranted to monitor the progress.

The associated factors of COVID-19 vaccine hesitancy found in the present study were largely consistent to those from previous studies [9, 14–16]. Okubo et al. revealed younger age, female, living alone, lower socioeconomic status, and presence of severe psychological distress were significantly associated with higher vaccine hesitancy rates [9]. Our findings add up to these findings by showing increased hesitancy among pregnant women. The Japan Society for Infectious Diseases in Obstetrics and Gynecology recommends that pregnant woman should not be excluded from vaccination programs [22, 23]. While there are currently limited data on the effect of pregnancy on the etiology of COVID-19 or the long-term safety of COVID-19 vaccines in pregnant women, vaccines are expected to protect expectant mothers from severe illness from COVID-19 [22, 23]. As getting vaccinated is a personal choice, it is important to facilitate equal, transparent, and timely dissemination of vaccine information to help all individuals with their decision making.

The present study is subject to several limitations. First, the internet-based sampling led to biased demographic distribution of the respondents. Although such bias was addressed by stratified analyses and multivariable adjustment, the results may not be representative of the entire Asmile users or the general population of Osaka. Specifically, the data collection was conducted on a voluntary basis that may have resulted in biased estimation of vaccine hesitancy. Given that the respondents were assumed to be more health-conscious and had higher internet literacy than those who did not participate in the survey, they possibly had keener perceptions on susceptibility, severity, barriers, and benefits regarding the COVID-19 vaccine. Since these perceptions contribute to determine the attitude toward the vaccine [7], vaccine hesitancy assessed in our study sample might have limited generalizability. Second, we were unable to conduct nuanced analysis on associations between socioeconomic status and public attitudes or behaviors regarding COVID-19 vaccination due to unavailability of such information in the Asmile survey. Continued assessment is warranted to understand the patterns and changes in those aspects to help plan and implement targeted interventions.

## Conclusion

During June 1–20, 2021, the majority (≥80%) of respondents in both sexes reported having received the COVID-19 vaccine or intending to receive one. Likelihood of reporting vaccine hesitancy was higher among women and younger, non-full-time working or unemployed, or pregnant individuals. Coordinated efforts are needed through effective communications and community-based interventions to ensure accessible vaccine information resources and healthcare services.

## Abbreviations

aPR: Adjusted prevalence ratio
CI: Confidence interval
COVID-19: Coronavirus disease 2019
